# First-Principles Study of Sr_2–*x*_La_*x*_FeO_4_ and Comparison with Sr_2_FeO_4‑δ_: The Nature of the Positively Charged Defect Determines the Character of the Electronic Structure

**DOI:** 10.1021/acs.jpcc.5c08764

**Published:** 2026-07-28

**Authors:** Yuri A. Mastrikov, Denis Gryaznov, Andrew Chesnokov, Guntars Zvejnieks, Maksim Sokolov, Maija M. Kuklja, Rotraut Merkle, Eugene Kotomin

**Affiliations:** † Institute of Solid State Physics, 487441 University of Latvia, Ķengaraga Street 8, Riga LV-1063, Latvia; ‡ University of Maryland College Park, College Park, Maryland 20742, United States; § University of Duisburg-Essen, Universitätsstraße 2, D-45141 Essen, Germany; ∥ Max Planck Institute for Solid State Research, Heisenbergstraße 1, D-70569 Stuttgart, Germany

## Abstract

Lanthanum doping of the first-order Ruddlesden–Popper (RP) phases Sr_2–*x*_La_*x*_FeO_4_ (0 ≤ *x* ≤ 1) is investigated by density functional theory (DFT) calculations. Excess volume and excess energy of this solid solution series are calculated. The evolution of the electronic structure with increasing La content is discussed based on the calculated Fe magnetic moment. It is found that for the same average Fe oxidation state, the electronic structure in Sr_2–*x*_La_*x*_FeO_4_ displays a more delocalized character than in corresponding Sr_2_FeO_4‑δ_ composition. This is attributed to the fact that oxygen vacancies V_O_^••^ which are formed under Fe–O bond breaking (and which carry a doubly positive charge) represent a stronger local distortion than singly charged substitutional La_Sr_^•^ dopants.

## Graphical abstract

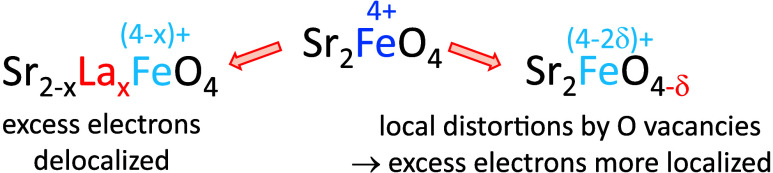

## 1 Introduction

The group of strontium ferrate perovskites and perovskite-related oxides is a fascinating material class. On one hand, it is interesting from a fundamental point of view, because different geometrical and electronic structures have very close energies so that delicate interactions finally decide which one becomes most stable. For the first-order Ruddlesden–Popper (RP) phase Sr_2_FeO_4_, magnetic structure measurements indicated a symmetry reduction from the tetragonal phase,^1^ and computational investigations also identified a symmetry reduction to orthorhombic and interpreted it as a Jahn–Teller (JT) distortion.^2,3^ In the second-order RP phase Sr_3_Fe_2_O_7_, the energy difference between a charge-ordered state (with Fe^3+^ and Fe^5+^) and a JT-distorted state is on the order of only 34 meV/formula unit, as found by the hybrid r²SCANh calculations.^4^

Overall, the electronic structure of Sr_*n*+1_Fe_*n*_O_3*n*+1 ± δ_ RP phases has a more localized character than that of the simple perovskite SrFeO_3‑δ_ (indicated, e.g., by the fact that the vacancy formation energy is almost constant in Sr_2_FeO_4‑δ_,^2^ while it strongly increases with increasing δ in SrFeO_3‑δ_ ^5^). This is to some degree simply related to the transition from a 3- to a 2-dimensional system. However, the comparison of Sr_2_FeO_4‑δ_ and Sr_2–*x*_La_*x*_FeO_4_ in the present investigation demonstrates that also the nature of the introduced positively charged defect (oxygen vacancies, or La^3+^ substituting for Sr^2+^) plays an important role. These defects not only decrease the average Fe oxidation state but also affect the character of the electronic structure. Introducing oxygen vacancies results in a rather localized character where—from a combination of Fe–O bond lengths and Fe magnetic moments—iron atoms with integer Fe^3+^ and Fe^4+^ oxidation states could be recognized.^1^ In the present study, we investigate the effects of La doping in Sr_2–*x*_La_*x*_FeO_4_, which will be shown to result in a more delocalized character.

The character of the Fe–O bonding is not only of fundamental interest but also directly affects the material’s properties regarding proton incorporation. Redox-active perovskites and perovskite-related materials serve as hole-oxygen vacancy mixed conducting or even proton-hole-vacancy triple conducting air electrode materials in solid oxide cells and protonic ceramic cells.^6–8^ Compared to *AB*O_3_ simple perovskites and *AA*’*B*_2_O_6_ or *A*_2_ *BB*’O_6_ double perovskites, RP phases comprise a higher fraction of *A*-site cations, which is expected to increase the overall basicity of the material. Further, the fraction of alkali earth (2+) and rare earth (3+) cations on the *A*-site affects the properties. Sr_2_FeO_4_ is an aristotype for the first-order RP phases utilized as mixed conducting electrode materials in ceramic electrochemical cells.^9–11^ While Sr_2_FeO_4_ itself needs to be prepared under high oxygen pressure,^12^ higher-order RP phases Sr_*n*+1_Fe_*n*_O_3*n*+1 ± δ_ or rare-earth doped compositions can be prepared under ambient conditions, e.g., Sr_3_Fe_2_O_7_ ^13,14^ or SrEu_2_Fe_2_O_7_.^15^ Besides Fe-based RP phases, also Co- and in particular Ni-based materials have been investigated as air electrode materials for protonic ceramic cells, e.g., (La,Sr_3_)­Co_1.5_Fe_1.5_O_10_,^16^ (Pr,Sr)_2_NiO_4_,^17^ (La,Sr)_2_NiO_4_,^18^ (Ba,Sr,La)_2_NiO_4_,^19^ or oxygen storage materials Sr_3_Fe_2–*x*_Ni_*x*_O_7‑δ_.^20^ Here, we concentrate on Fe-based Sr_2–*x*_La_*x*_FeO_4_, RP phases where we can compare with the preceding computational work with the same methodological approach for Sr_2_FeO_4‑δ_ and SrFeO_3‑δ_.^2,5,21^ While both oxygen vacancies and La dopants are positive defects (charge-compensated by a decrease of the average iron oxidation state), the present results indicate that they modify the electronic structure of the material in different ways. This comparison enhances the general understanding of the defect chemistry of mixed conducting oxides, particularly in delicate situations as encountered in the Ruddlesden–Popper oxides where the energetic difference between more or less localized electronic defects is small.

## 2 Method and Model

Doping Sr_2_FeO_4‑δ_ by La has been performed by substitution La_Sr_^•^. La concentration *x* has been varied from 0.125 up to 1.00. Varying *x* continuously, we minimize the possibility of technical errors in calculations and ensure the integrity of the results within the range of interest. The supercell of eight Sr_2_FeO_4_ formula units (56 atoms) is suitable for modeling a relatively low La concentration (*x* = 0.125). Only one inequivalent configuration of La_Sr_ is possible in a model supercell. For higher values of *x*, all possible spatial distributions of La_Sr_ have been investigated (Table S1) by means of the Supercell code.^22^ Screening of this large number of configurations proved necessary because the lowest energy configuration for a given La content in the supercell does not necessarily occur for the intuitively guessed arrangements with maximum La distances (cf. discussion of Figures 2 and S1). Note that representability of a particular configuration depends on its energy, multiplicity, and structural stability. Calculations have been performed by the computer code VASP.^23^ Effects of core electrons were emulated by PAW^24^ potentials (Table S2). Computational details are presented in Table 1. The Hubbard correction value *U*_eff_ = 4 eV has been chosen based on an extensive analysis of structural and electronic parameters of SrFeO_3_ and Sr_2_FeO_4_.^2^ In addition to that, the value has been considered optimal for similar (Ba,Sr)­FeO_3‑δ_ and (Pr,Sr)_2_FeO_4‑δ_ perovskites.^21,25,26^

**Table 1:** Main Computational Parameters

computer code	VASP 6.1.2
cutoff energy	520 eV
space group (SG), Settings	64, *Bbem*
transformation matrix from the crystallographic unit cell to the supercell	$$\left(\begin{array}{lll} 1 & 1 & 0\\ -1 & 1 & 0\\ 0 & 0 & 1 \end{array}\right)$$
*K*-points	Monkhorst–Pack^27^4 × 4 × 2
exchange–correlation functional	Perdew–Burke–Ernzerhof^28^
*U*_eff_	4.0 eV
residual external pressure	<1 kbar
forces on atoms	<0.02 eV/Å

Electron charge and magnetic moments were analyzed by the Bader method.^29^ Structure visualization was performed using the code VESTA.^30^

Considering one extra electron released to the system by each substitution of Sr^2+^ by La^3+^ (La_Sr_^•^), and two extra electrons released by an oxygen vacancy, the formal Fe^3+^/Fe ratio can be defined as follows:

1 $${Sr}_{2-x}{La}_{x}{Fe}_{1-\left(2\delta +x\right)}^{4+}{Fe}_{2\delta +x}^{3+}O_{4-\delta }$$

The high covalency of the Fe–O bonds in the system prevents a clear distinction between these two oxidation states of Fe. A direct charge calculation by the Bader method proved to be unreliable for assigning Fe oxidation states. Therefore, for oxidation state identification, we used magnetic moment combined with the lengths of Fe–O bonds, as proposed in ref 2.

## 3 Results

### 3.1 Excess Energy, Volume, and Spatial Distribution of La_Sr_^•^

The energetic stability of a complex material like Sr_2–*x*_La_*x*_FeO_4‑δ_ depends on defect (*A*-cation substitution, oxygen vacancy/interstitial) concentration as well as on their spatial distribution. Oxygen defects have been studied in detail in ref 2 In the present work, we focus on La_Sr_^•^ substitution, preserving the oxygen stoichiometry at δ = 0, except for *x* = 0.125 (discussed below). The energetic stability of Sr_2–*x*_La_*x*_FeO_4_ was calculated as excess energy relative to the linear interpolation between the La-free composition (*x* = 0) and the lowest energy state for *x*_ref._ = 1.00 (Table S3 and Figure S1d).

2 $$E_{excess}=E_{x}-\left(E_{x=0}+\left(E_{\min\;x=1}-E_{x=0}\right)x\right)$$

The nonzero excess energies in Figure 1a indicate moderate deviations from an ideal solution. The negative excess energy values indicate an overall driving force for mixing between Sr_2_FeO_4_ and SrLaFeO_4_ (the distribution of La and Sr within the cell may still have a preference for the La segregation, see later). Comparison with other perovskite-type materials (Table 2) indicates that a smaller size mismatch and more flexibility in the structural and chemical bonding properties (e.g., presence of more loosely bound rocksalt layers in RP phases) favor the formation of stable solid solutions. The rocksalt AO layer in the RP phase offers even more flexibility to accommodate differently sized *A*-cations (for RP phases of higher orders even with crystallographically different *A*-sites).

**Figure 1:**
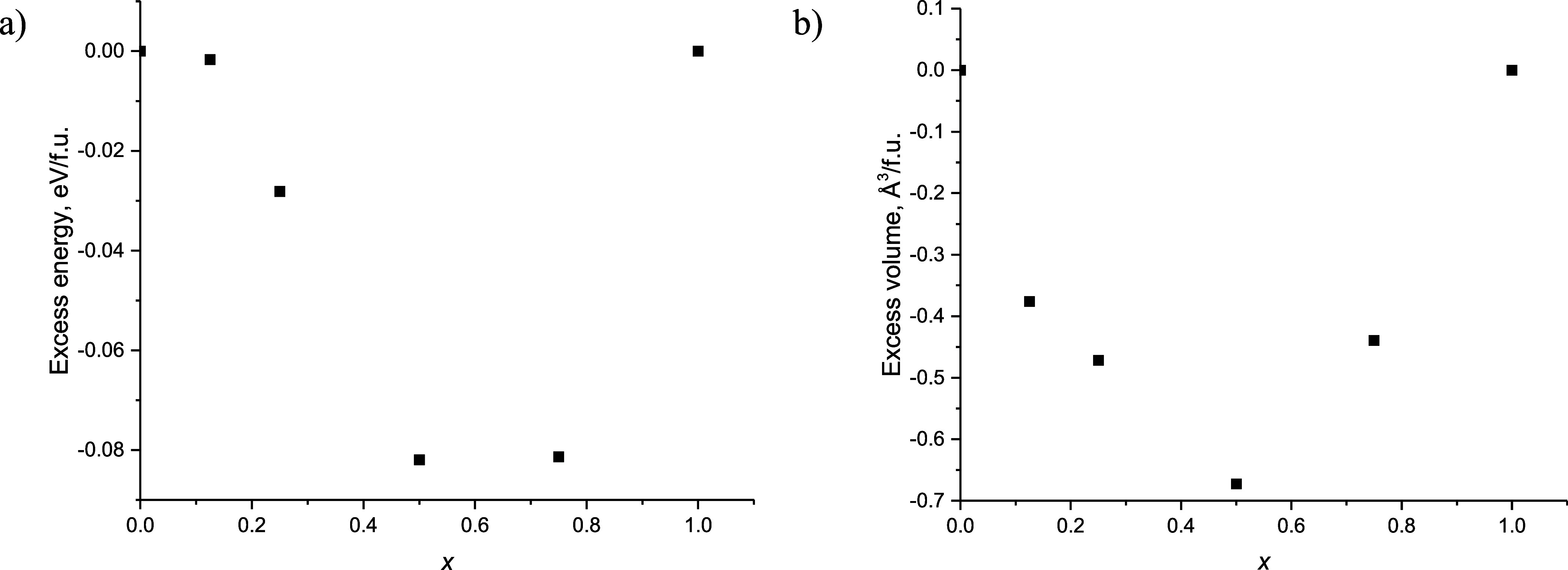
(a) Excess energy (per formula unit) of Sr_2–*x*_La_*x*_FeO_4_. (b) Excess volume (per formula unit) of Sr_2–*x*_La_*x*_FeO_4_. The data correspond to the configurations with the lowest total energy for each La content.

**Table 2:** Excess Energy for Various Composition Changes in Perovskite-Related Structures: *A*_2_ *B*O_4_ and *AB*O_3_

material	structure	mixing cation, size difference, Å	variation	excess energy	source
Sr_2–*x*_La_*x*_FeO_4_	1st order RP	*A* 0.08	atomic radius, oxidation state of A- and B-cations	negative	this study
La_1–*x*_Sr_*x*_CoO_3_	perovskite	*A* 0.08			^31^
La_1–*x*_Sr_*x*_MnO_3_	perovskite	*A* 0.08			^32^
Ba_1–*x*_Sr_*x*_TiO_3_	perovskite	*A* 0.17	atomic radius	positive	^33^
BaFe_1–*x*_Ce_*x*_O_3_	perovskite	*B* 0.28			^34^

Interestingly, the excess energy curve of Sr_2–*x*_La_*x*_FeO_4_ has an asymmetric shape. Compositions with intermediate La content *x* = 0.5 and *x* = 0.75 have the most negative excess energies, i.e., the minimum is shifted toward higher La contents. Also, the volume shows moderate deviations from the ideal behavior Figure 1b, but in contrast to the excess energy, the excess volume shows an approximately symmetrical shape. The absolute molar volume increases with increasing La substitution despite La^3+^ (1.36 Å) having a smaller Shannon radius than Sr^2+^ (1.44 Å, both in 12-fold coordination), in line with experimental data.^35^ This volume expansion is caused by a decrease in the average Fe oxidation state and an increase in the corresponding Fe ionic radius. The volume expansion between Sr_2_FeO_4_ and SrLaFeO_4_ amounts to 2.7%, which is significantly larger than the respective expansion by 1% between Sr_2_FeO_4_ and Sr_2_FeO_3.5_ (also changing from average Fe^4+^ to Fe^3+^).^2^ This results from the fact that in Sr_2–x_La_*x*_FeO_4_ the Fe reduction is not accompanied by removal of oxygen ions.

Figure 2 shows that for each La content the lowest energy is found for the configurations with the shortest average La–O distance (configurations shown in Figure S1). These arrangements lead to the most beneficial La^3+^–O^2–^ electrostatic interactions. A more detailed analysis (Figure S2) indicates the sensitivity for certain detailed structural features (which together finally determine the average La–O distance). Figure S2a demonstrates that for a given *x*, a larger number of La_Sr_^•^–La_Sr_^•^ bonds (1NN) across the perovskite layer slightly increases the energy. A larger number of 2NN La_Sr_^•^–La_Sr_^•^ bonds lowers the energy (Figure S2b,e,h). Both criteria together correspond to accumulating La within the same rocksalt layer (but distributed over both AO planes of that layer). If several La_Sr_^•^ are located within the same AO plane, then the configurations with more 3NN pairs are more favorable (Figure S2c). This can be traced back to the fact that this arrangement allows for shorter La–O and longer Sr–O distances reflecting their size as well as charge difference (Figure 3b), in contrast to the case of Figure 3c, where the topology enforces equal—but energetically less favorable—La–O and Sr–O lengths. So, the spatial arrangement does not simply follow the principle of maximizing or minimizing the distance between La_Sr_^•^ substitutions which carry a relative positive charge compared to Sr.

**Figure 2:**
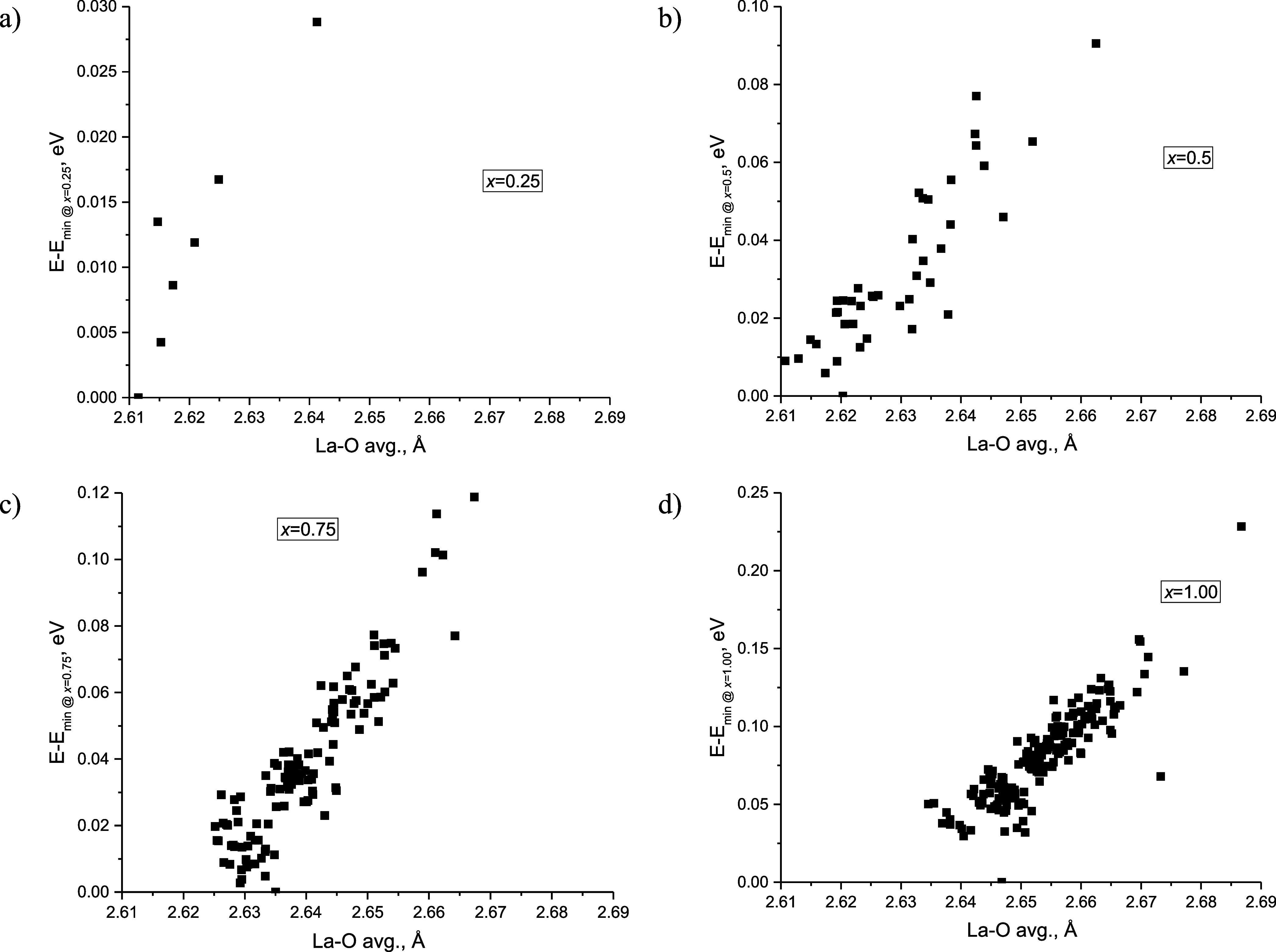
Energy per f.u. relative to the lowest value for each value of *x* depending on the averaged La_Sr_^•^–O distance, which is obtained by averaging over the nine La–O bonds around La and all La_Sr_^•^ cations.

**Figure 3:**
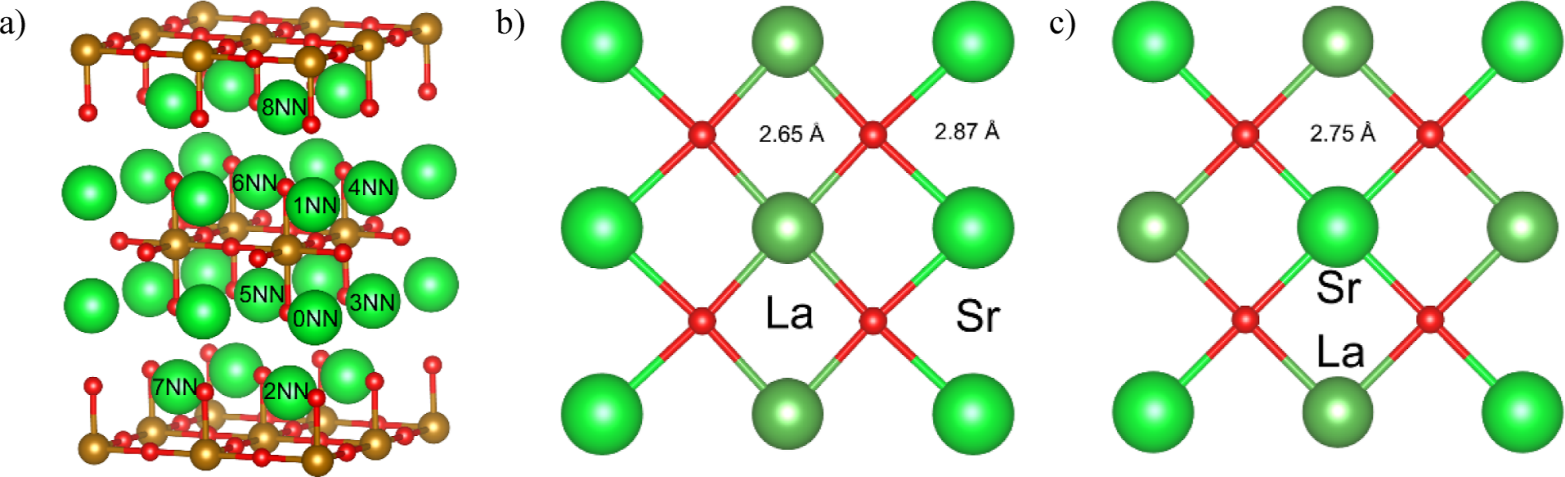
Nearest neighbors (NN) of the A-cation sublattice in the Sr_2_FeO_4_ RP 1st-order perovskite structure in an 8-formula unit supercell (a). (Note that the periodic boundary conditions are neglected solely for showing the 7NN). La_Sr_ cations in the Sr_1.75_La_0.25_FeO_4_ structure as 3NN (b) and 5NN (c).

We also briefly explored the energetics of oxygen-deficient composition Sr_1.875_La_0.125_FeO_3.875_ (Table S4). Since La_Sr_^•^ and V_O_^••^ are both positive defects, it is not surprising to find a moderate positive interaction energy (Figure S4), in the range from 0.2 to 0.8 eV. Since the interaction energy depends only slightly on the actual La_Sr_^•^–V_O_^••^ distance, the presence of V_O_^••^ is not expected to significantly change the relative stability of different La_Sr_^•^–La_Sr_^•^ configurational arrangements for the higher La concentrations. Just like in the case of Sr_2_FeO_4_,^2^ V_O_^••^ forms preferentially in the FeO_2_ planes. The lowest V_O_^••^ formation energies in Sr_1.875_La_0.125_FeO_4_ are only slightly higher than in Sr_2_FeO_4_ (2.60 and 2.35 eV for Sr–V_O_^••^-Fe and Fe–V_O_^••^-Fe, respectively), which is in line with the fact that for Sr_2_FeO_4‑δ_ only a minor variation of V_O_^••^ formation energy with an iron oxidation state has been observed.^2^

### 3.2 Identification of Fe Oxidation States in Sr_2–x_La_*x*_FeO_4_

The high degree of covalency of the Fe–O bonds prevents a clear distinguishing between the 4+ and the 3+ oxidation states of Fe. As discussed in more detail in,^2^ the Fe–O bonds in the simple perovskite SrFeO_3_ have a higher charge transfer from the oxide ions to iron and more delocalized character than in Sr_2_FeO_4_ with the same formal Fe^4+^ oxidation state. Nevertheless, the Fe–O bonds in Sr_2_FeO_4_ are still far from purely ionic, and thus, the question whether fixed Fe oxidation states can be assigned is not trivial (attempting to use the Bader charges yields inconsistent results). As proposed in,^2^ the Fe magnetic moment combined with the lengths of Fe–O bonds can serve as an indirect indicator of the oxidation state (Figure S5). Already for *x* = 0.125 (one extra electron in a supercell), we observed quite a broad distribution of the magnetic moments (Figure 4a); it is not possible to recognize a single Fe cation with a clear 3+ oxidation state where the excess electron is localized (the data points at high magnetic moment ≈3.84 μ_B_ represent a total of five (2NN + 4 × 1NN) Fe atoms, Table S5). Reference cells which contain only Fe^3+^ have much higher magnetic moments: (i) in SrLaFeO_4_, the moments amount to 4.25–4.30 μ_B_, (ii) in Sr_2_FeO_4‑δ_, the moments cover a larger range of 4.05–4.25 μ_B_ (larger moments for larger δ ^2^). In Figure 4a, the corresponding ranges are indicated in dark gray (comparison with SrLaFeO_4_) and light gray (lower end interpolated according to the La content between SrLaFeO_4_ and Sr_2_FeO_4‑δ_ with low δ). None of the Fe in Sr_1.875_La_0.125_FeO_4_ comes close to the 3+ range. Some Fe are in the lower part of the Fe^4+^ reference zone (3.65–3.8 μ_B_ for Sr_2_FeO_4_ and Sr_2_FeO_3.875_,^2^ beige area), while others attain values around 3.85 μ_B_. The latter are regarded as a result of delocalization of the excess electron over several iron cations.

**Figure 4:**
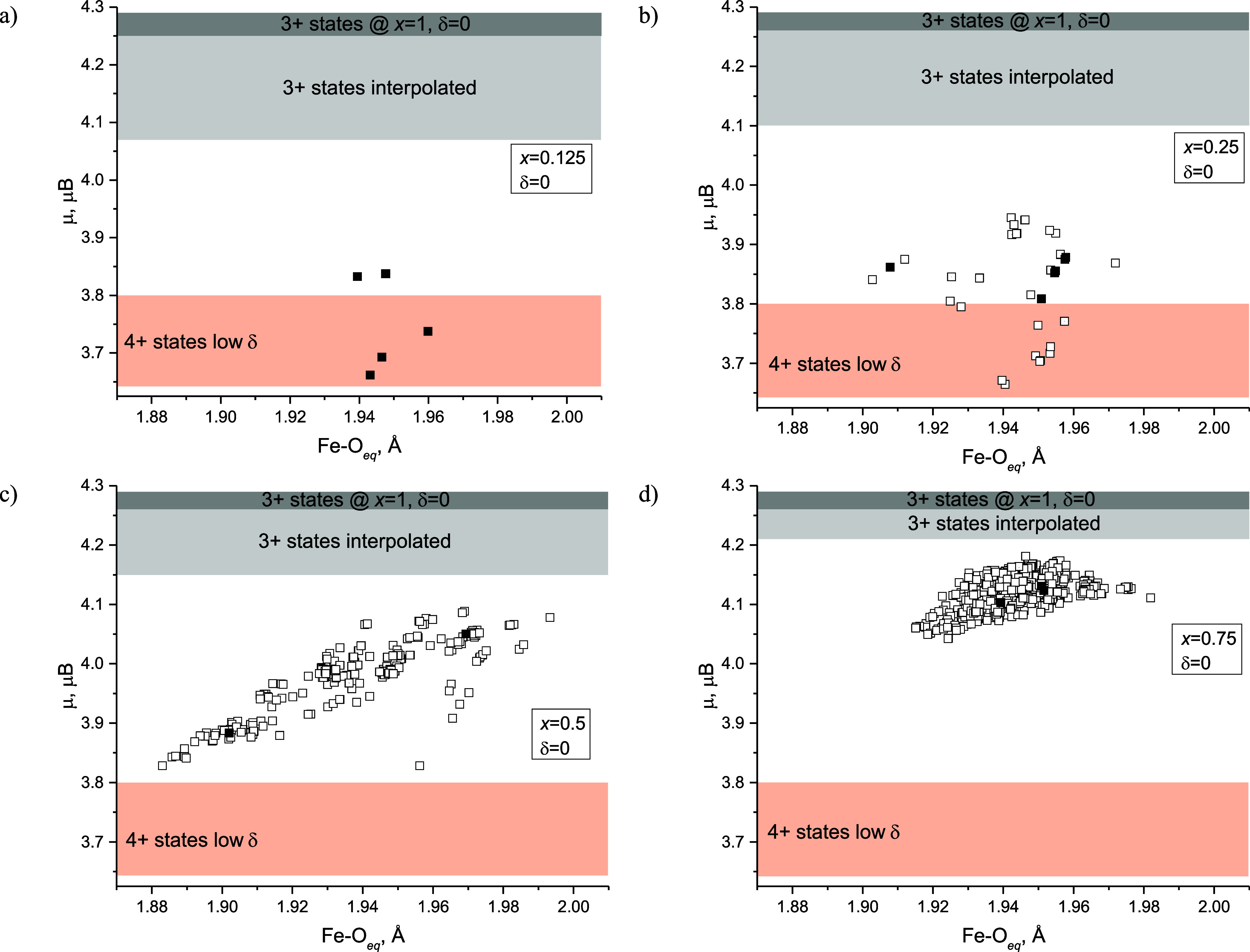
Magnetic moment as a function of averaged Fe–O_eq_ distance (average of the Fe–O_eq_ around each individual Fe in the supercell) for δ = 0 and (a) *x* = 0.125, (b) *x* = 0.25, (c) *x* = 0.5, and (d) *x* = 0.75. Solid symbols denote the values for the lowest energy configurations. The shaded areas indicate the zones where integer Fe^3+^ and Fe^4+^ oxidation states were assigned for reference configurations. Figure S3 shows the quite similar plots with Fe–O_ap_ and Fe–O (all) on the horizontal axis.

For *x* = 0.25, the situation is closely related (Figure 4b). The configuration with the lowest energy (solid symbols) has only Fe with moments higher than the 4+ reference, i.e., the two excess electrons are homogeneously distributed among all Fe in the cell. However, there are configurations at only slightly higher energy with some Fe^4+^ besides other Fe with decreased oxidation state. For *x* = 0.5, we see a broad distribution of moments, not touching 3+ and only barely touching the 4+ reference values (Figure 4c). At a high La content of *x* = 0.75 (average Fe oxidation state 3.25+), the distribution further shifts to higher μ_B_ values and becomes again narrower (Figure 4d). Finally, for *x* = 1 (all iron Fe^3+^), all moments are within 4.25–4.3 μ_B_. So, for most of these cases, it is not possible to assign integer 3+ and 4+ oxidation states, and even if some Fe fall into the region of the 4+ state, the excess electron(s) may still be distributed over several other Fe cations.

For *x* = 0.125 (the composition with the highest tendency to form localized states), we ran extended test calculations attempting to obtain a fully localized polaron (a single Fe^3+^) by using starting geometries with deliberately distortion Fe–O bond lengths primarily within the DFT + *U* methodology. However, Table S6 demonstrates that such a solution was not obtained, and further that the energies of partially localized and fully delocalized states are nearly indistinguishable within the accuracy of the method (∼4 meV).

To further check the trend of the increasingly delocalized character of the electronic structure with high La content, we also used the HSE06 ^36^ functional to calculate the configurations with the lowest energy (Figure S1) for various La contents (details are given in the Supporting Information). Figure S6 compares the data for the lowest-energy configurations obtained with DFT + *U* and HSE06. The overall trend of increasing delocalization of the excess electron(s) with increasing La content is observed for both methods. For HSE06, even for the lowest La content, no single Fe^3+^ is found (the maximum magnetic moment remains much below the value expected for Fe^3+^, and four Fe have an increased magnetic moment, i.e., the excess electron is shared between the 4 × 1NN Fe). A comparison of the electronic density of states (DOS) calculated using DFT + *U* and the HSE approach is given in Figures S7–S9. Also, here the main trends with varying La content agree well. This is encouraging to see, because HSE calculations are more computationally demanding and more delicate to properly converge.

### 3.3 Comparison with Sr_2_FeO_4_

A direct comparison with the earlier studied Sr_2_FeO_4‑δ_^2^ can be made for combinations of δ and *x* that yield identical average Fe oxidation states (i.e., δ_equiv_ = *x*/2). Considering one extra electron released to the system by the La_Sr_^3+^ cation and two extra electrons released by oxygen vacancy, formally, the Fe^3+^/Fe^4+^ concentrations can be defined as follows:

3 $${Sr}_{2-x}{La}_{x}{Fe}_{1-\left(2\delta +x\right)}^{4+}{Fe}_{2\delta +x}^{3+}O_{4-\delta }$$

Particularly, we can compare two cases *x* = 0.25 and *x* = 0.5 for δ = 0 with δ = 0.125 and δ = 0.25 for *x* = 0, respectively. Figure 5a,b compares the situation for the averaged Fe^3.75+^ state. In Sr_2_FeO_3.875_, one can recognize several Fe which are identified as 4+ based on magnetic moments (3.65–3.8 μ_B_) and Fe–O bond length distribution^2^ and others attributable as Fe^3+^. This is in pronounced contrast to the more continuous moment distribution for Sr_1.75_La_0.25_FeO_4_, where in particular no Fe reaches the Fe^3+^ zone. A similar situation is found for Fe^3.5+^ in Figure 5c,d, where in Sr_2_FeO_3.75_ clearly a large number of Fe^3+^ and Fe^4+^ can be recognized (with larger δ, the range attributable to 4+ expands up to 3.8 μ_B_,^2^ shaded light red). In contrast, Sr_1.5_La_0.5_FeO_4_ presents a very smooth moment distribution which does not reach the 3+ and hardly touches the 4+ zone. So, the overall conclusion is that Sr_2–x_La_*x*_FeO_4_ presents a much more delocalized situation than Sr_2_FeO_4‑δ_. Analogously, experimental Mössbauer spectra for Sr_2–*x*_La_*x*_FeO_4_ samples with 0.7 ≤ *x* ≤ 1.0 were fitted with a significant fraction of a mixed 3+/4+ oxidation state.^37^

**Figure 5:**
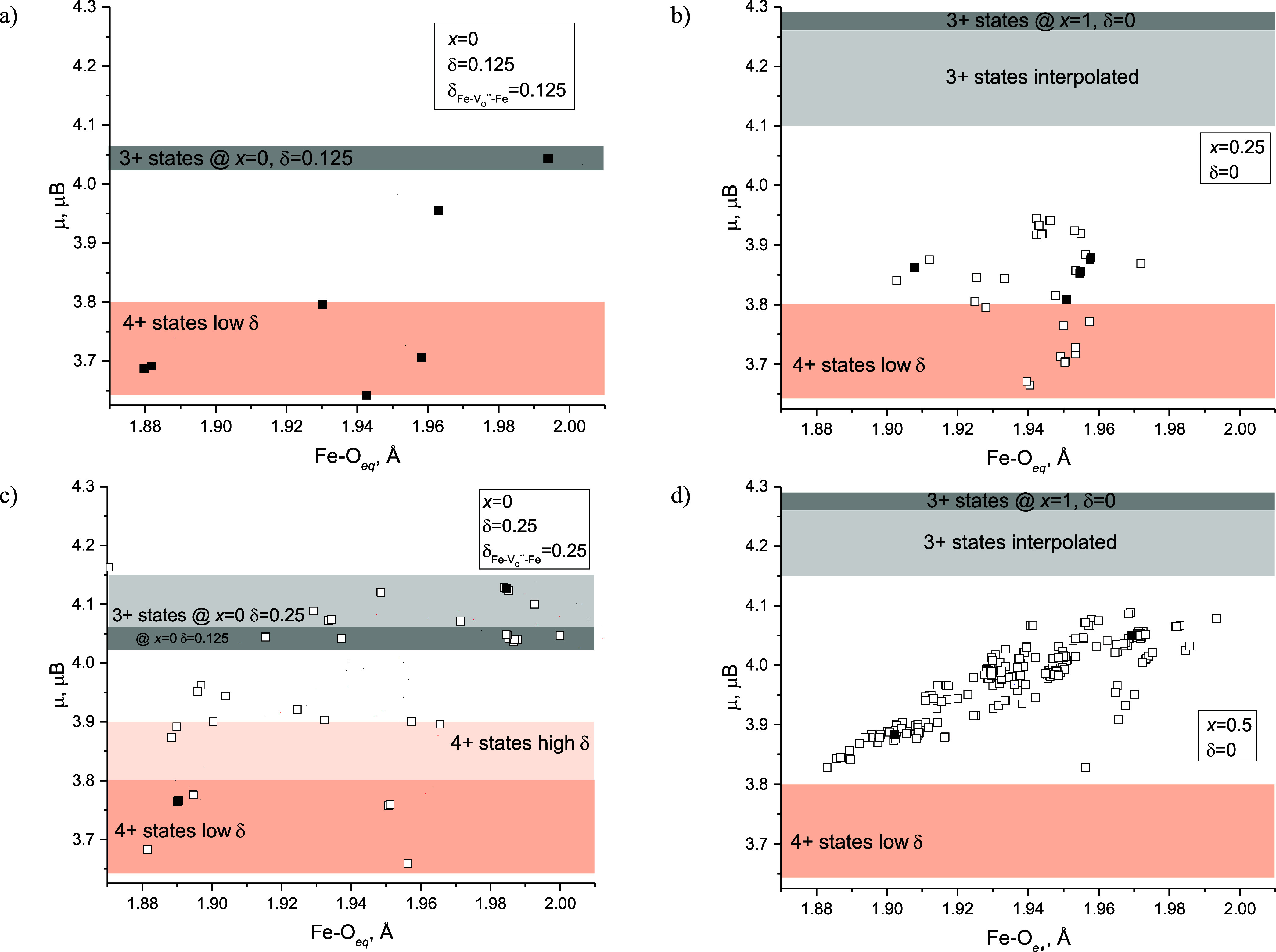
Magnetic moment as a function of averaged Fe–O_eq_ for average Fe^3.75+^: (a) *x* = 0; δ = 0.125; and (b) *x* = 0.25 δ = 0. For average Fe^3.5+^: (c) *x* = 0; δ = 0.25; and *x* = 0.5; δ = 0. For the Sr_2_FeO_4‑δ_ cells with δ > 0, only the energetically more stable Fe–V_O_^••^–Fe vacancy configurations are considered. Solid symbols denote the values for the lowest energy states.

This difference can be rationalized from the following considerations. The presence of oxygen vacancies in Sr_2_FeO_4‑δ_ constitutes a much stronger distortion than the substitution of some Sr^2+^ by La^3+^ (although both defects lead to a decrease of the average Fe oxidation state). An oxygen vacancy causes broken Fe–O bonds, which leads to significant structural relaxations. Further, it was shown in ref 2 that the presence of an oxygen vacancy affects the electronic structure of iron in a way similar to a JT distortion (expanding two axial Fe–O bonds is not too different from completely removing one O ligand). This can stabilize localized Fe^4+^ states, and then in turn also the states accommodating the excess electrons caused by the oxygen deficiency acquire a more localized character. In contrast, such a specific driving force for forming localized Fe^4+^ states is absent in Sr_2–*x*_La_*x*_FeO_4_. An additional reason for the more localized character of Sr_2_FeO_4‑δ_ might be the fact that oxygen vacancies are doubly positively charged defects (while substitutional La^3+^ has only a single relative positive charge compared to Sr^2+^), which is expected to cause more pronounced local distortions. The strong effects of oxygen vacancies and related structural distortions have also been observed in the SrFeO_3‑δ_ simple perovskites where the electronic conductivity changes from metallic character for the cubic δ = 0 phase to polaron hopping in vacancy-ordered noncubic phases for δ > 0.03.^38^

So, the material class of strontium ferrate perovskite and Ruddlesden–Popper phases is an intriguing example of transition-metal oxides where materials with more or less pronounced delocalized character of the electronic structure are quite close in energy. Correspondingly, small changes suffice to switch the system into one or the other direction. These may be the lattice parameter expansion in the Sr_1–*x*_Ba_*x*_FeO_3_ series which induces a change from delocalized character, the absence of JT distortion, and metallic-type conductivity to a more localized character with polaron hopping for Sr_0.5_Ba_0.5_FeO_3_ (or possibly already for lower Ba contents).^21^

The differences of the Fe–O bonding character are not only of fundamental interest. Preceding DFT investigations have shown that the propensity of oxides for proton uptake by water incorporation is closely coupled to the material’s proton affinity and further related to the character of the O 2p states.^39^ Within a BaFeO_3‑δ_ supercell, O atoms opposite to an oxygen vacancy exhibit an increased covalency of the significantly shortened Fe–O bond with a decreased center of gravity of the O 2p states and correspondingly are less favorable for protonation.^25^ It remains to be investigated whether a material such as Sr_2_FeO_4‑δ_ with distinctly different oxygen atoms (i.e., some strongly preferred protonation sites), or Sr_2–*x*_La_*x*_FeO_4_ with more equivalent oxygens yields a higher total proton uptake (for comparable average Fe oxidation state). However, in the former, quite pronounced proton trapping effects are anticipated, which will be detrimental for a long-range proton transport.

## 4 Conclusions

This computational investigation of the Sr_2–*x*_La_*x*_FeO_4_ first-order RP phase indicates that the La/Sr configurations with the shortest average La–O bond length yield the lowest total energy, and that excess energy as well as excess volume in the Sr_2_FeO_4_–SrLaFeO_4_ solid solution series are negative. While La substitution decreases the average (formal) Fe oxidation state in Sr_2–*x*_La_*x*_FeO_4_, it is not possible to identify any localized Fe^3+^ states based on magnetic moments. This is in contrast to oxygen-deficient Sr_2_FeO_4‑δ_ where localized Fe^3+^ species had been identified. This indicates that—for the same average Fe oxidation state—the electronic structure exhibits a more delocalized character in Sr_2–*x*_La_*x*_FeO_4_ compared to Sr_2_FeO_4‑δ_. The different behavior is attributed to oxygen vacancies V_O_^••^ (with doubly positive charge and breaking of Fe–O bonds) exerting a stronger local distortion than singly charged La_Sr_^•^ and thus shifting the delicate energy balance between delocalized and more localized states toward the latter. Such differences in the electronic structure also modify the basicity of individual oxide ions and may thus affect the energetics of proton incorporation. This remains to be investigated in the future.

## Supplementary Material



## Notes

Inequivalent La_Sr_^•^ configurations and energetics; PAW–PBE core–electron effects; relative stability with respect to linear interpolation; La_Sr_^•^–La_Sr_^•^ clustering effects and A–O coordination scheme; defect interaction energies for V_O_^••^–La_Sr_^•^ complexes; Fe–O bond-length–magnetic moment correlations; Fe magnetic moments and defect-induced localization; structural and electronic parameters from HSE06 and DFT + *U*; and electronic density of states for Sr_2–*x*_La_*x*_FeO_4_ (PDF)
