# Supplementary material for: First-Principles Study of Sr2–x La x FeO4 and Comparison with Sr2FeO4‑δ: The Nature of the Positively Charged Defect Determines the Character of the Electronic Structure
Source: J Phys Chem C Nanomater Interfaces. 2026 Jul 28;130(31):11001–8. doi: 10.1021/acs.jpcc.5c08764 (PMC13455106; doi:10.1021/acs.jpcc.5c08764)
Supplement: Supplementary file 1 [file jp5c08764_si_001.pdf]

Supporting Information

First principles study of $Sr_{2-x}La_xFeO_4$ and comparison with $Sr_2FeO_{4-\delta}$: the nature of the positively charged defect determines the character of the electronic structure

Yuri A. Mastrikov[*1,2], Denis Gryaznov[*1], Andrew Chesnokov[1], Guntars Zvejnieks[1], Maksim Sokolov[1,3], Maija M. Kuklja[2], Rotraut Merkle[*4], Eugene Kotomin[1,4]

[1]Institute of Solid State Physics, University of Latvia, Ķengaraga Street 8, Rīga, LV-1063, Latvia
[2]University of Maryland College Park, College Park, Maryland 20742, United States
[3]University of Duisburg-Essen, Universitätsstraße 2, D-45141, Essen, Germany
[4]Max Planck Institute for Solid State Research, Heisenbergstraße 1, D-70569, Stuttgart, Germany

[*]corresponding authors: yuri.mastrikov@cfi.lu.lv; gryaznov@cfi.lu.lv; r.merkle@fkf.mpg.de

Table of Contents

Table S1;
Table S2;
Figure S1;
Table S3;
Figure S2;
Figure S3;
Figure S4;
Table S4;
Defect interaction and formation energy analysis;
Figure S5;
Table S5;
Exploring a possible Fe^{3+} polaron formation depending on the starting conditions;
Table S6;
Table S7;
Effects of DFT+U versus HSE06 functional on the degree of localization;
Table S8;
Figure S6;
Fe magnetic moment distributions for different La contents; dependence on Fe–O_{eq} distance: comparison of HSE06 and DFT+U results;
Electronic density of states (DFT+U and HSE06) for $Sr_{2-x}La_xFeO_4$: electronic structure evolution with La content;
Figure S7;
Figure S8;
Figure S9.

Table S1. Number of inequivalent configurations (spatial distribution of $La_{Sr}^{\bullet}$) on La concentration x.

x	Number of inequivalent configurations
0	1
0.125	1
0.25	7
0.5	42
0.75	122
1	181

Table S2. Projector Augmented-Wave (PAW) Perdew-Burke-Ernzerhof (PBE)[40] potentials for core electrons effect.

Potential	Valence electrons	E_{cutoff}, eV
Sr	$4s^2 4p^6 5s^2$	229.353
La	$5s^2 6s^2 5p^6 5d^1 4f^0$	219.292
Fe	$3d^7 4s^1$	267.883
O	$2s^2 2p^4$	400.000

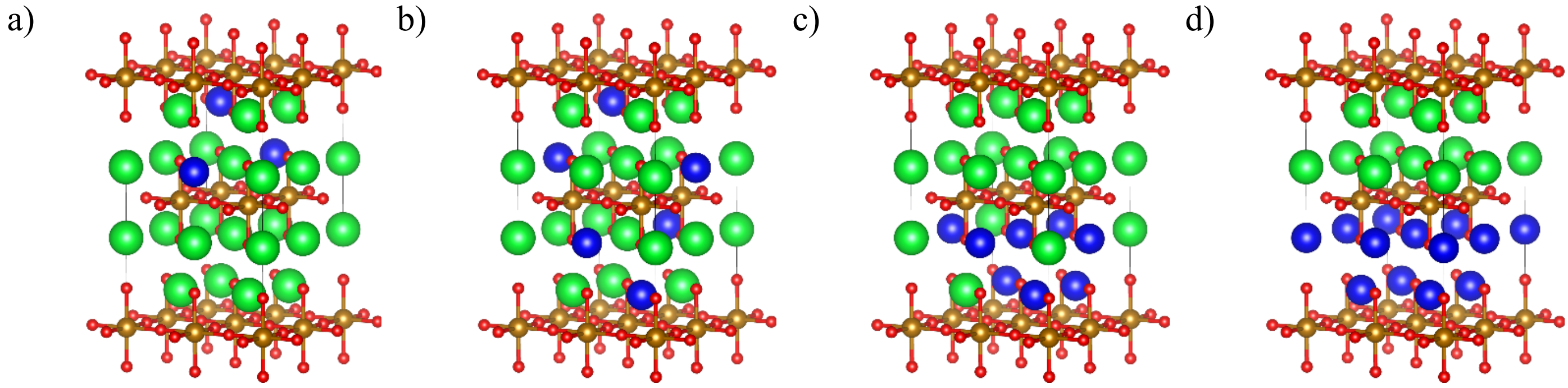

Figure S1. The lowest energy configurations ($La^{\bullet}_{Sr}$ spatial arrangement) for $x = 0.25$ (a); 0.5 (b); 0.75 (c) and 1(d). Sr - green, La - blue.

Table S3. The lowest energy, relative to the linear interpolation between $E_{x=0}$ and $E_{x=1\ min}$ according to Eq. 2 of the main text.

x	$E_{x\ min}$, eV/f.u.	Cell volume, Å^3/f.u.	a_0, Å	b_0, Å	c_0, Å	VASP free energy*, eV
0.000	0.000	95.43	7.823	-	12.475	-357.16
0.125	-0.002	95.37	7.787	7.791	12.577	-363.08
0.250	-0.028	95.60	7.766	-	12.680	-369.19
0.500	-0.082	96.04	7.735	-	12.840	-381.43
0.750	-0.081	96.91	7.738	-	12.960	-393.22
1.000	0.000	97.99	7.808	-	12.859	-404.38

* The energies, as printed by the code VASP, relevant only for particular computational settings.

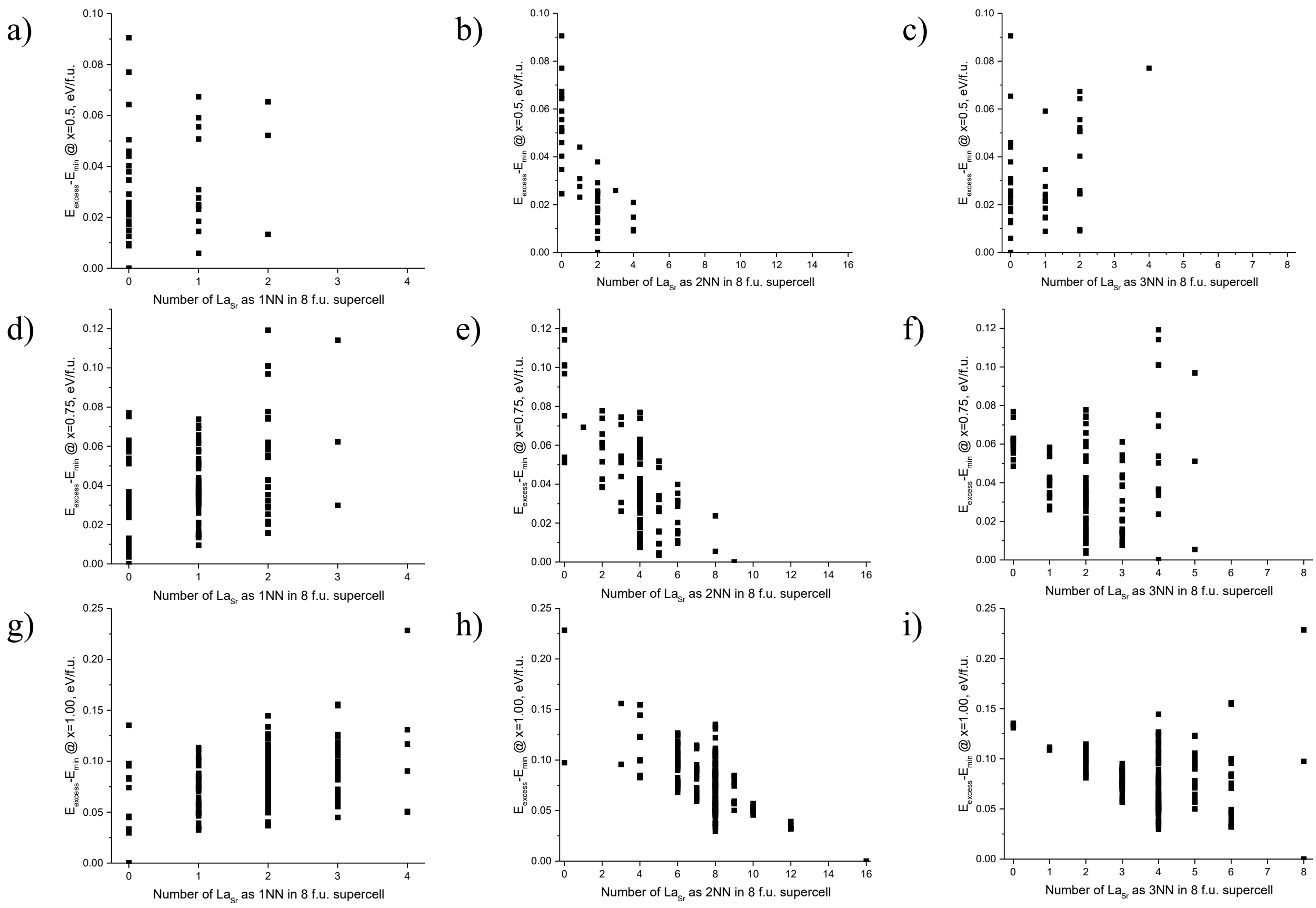

Figure S2. Relative energy of $Sr_{2-x}La_xFeO_4$ (Eq. 2) as a function on the number of La_{Sr}-La_{Sr} bonds of particular length (NN) in supercell (Figure 3a).

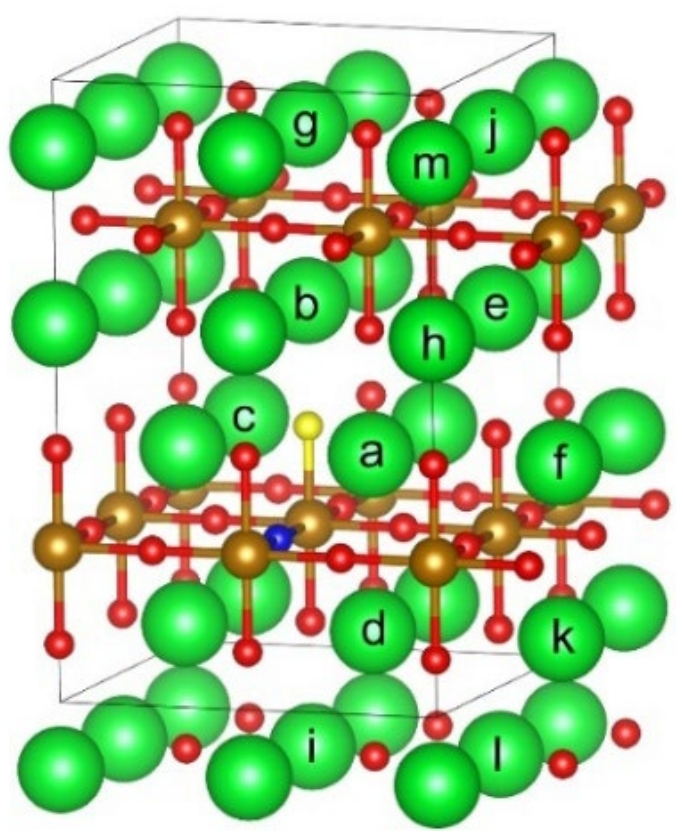

Figure S3. The A-O_{ap} Nearest Neighbours and A-O_{eq} NN scheme. Fe = brown, O = red, A (Sr or La) = green. Yellow – O_{ap} and blue = O_{eq} are the reference anions. For indexing of A-cations and absolute distances see Table S 4.

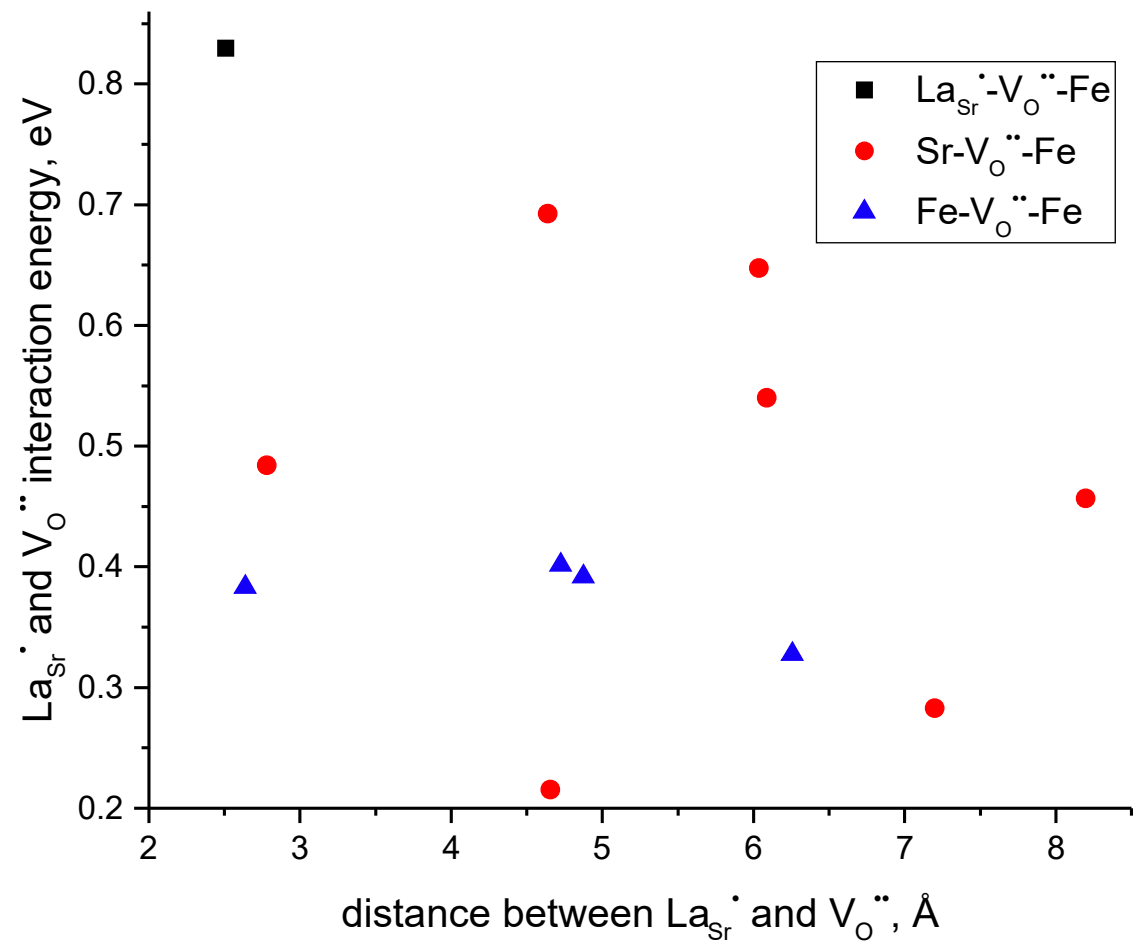

Figure S4. Plot of $V_O^{\bullet\bullet}$-$La_{Sr}^{\bullet}$ interaction energy as a function of $V_O^{\bullet\bullet}$-$La_{Sr}^{\bullet}$ distance, for two types of oxygen vacancies Sr-$V_O^{\bullet\bullet}$-Fe and Fe-$V_O^{\bullet\bullet}$-Fe. $La_{Sr}^{\bullet}$-$V_O^{\bullet\bullet}$-Fe vacancy is possible within the model cell and at x = 0.125 for the single case – 1NN.

Table S4: Energies for $Sr_{1.875}La_{0.125}FeO_{3.875}$ (one $V_O^{\bullet\bullet}$, and one $La_{Sr}^{\bullet}$ dopant in the supercell) cf. Fig. S 3.

Sr-$V_O^{\bullet\bullet}$ -Fe	NN	$V_O^{\bullet\bullet}$-$La_{Sr}^{\bullet}$ distance, Å	$V_O^{\bullet\bullet}$-$La_{Sr}^{\bullet}$ interaction energy**, eV	$V_O^{\bullet\bullet}$ formation energy, eV	VASP free energy***, eV
b*	1	2.51	0.83	3.21	-355.76
a,c	2	2.78	0.48	2.87	-356.10
d	3	4.64	0.69	3.08	-355.89
e	4	4.66	0.22	2.60	-356.37
g	5	6.04	0.65	3.03	-355.94
h	6	6.09	0.54	2.92	-356.05
f****	7	6.20	-	-	-
i****	8	6.42	-	-	-
j	9	7.20	0.28	2.67	-356.30
k****	10	7.23	-	-	-
l****	11	7.52	-	-	-
m	12	8.20	0.46	2.84	-356.13
Fe-$V_O^{\bullet\bullet}$-Fe					
a,d	1	2.64	0.38	2.41	-356.56
c	2	4.73	0.40	2.43	-356.54
b,i	3	4.87	0.39	2.42	-356.55
f,k****	4	6.14	-	-	-
e,h,l	5	6.26	0.33	2.35	-356.62

*The only case of (La-$V_O^{\bullet\bullet}$-Fe) vacancy (1NN).
**The interaction energy is calculated relative to $Sr_{1.875}La_{0.125}FeO_4$ (-368.08eV), $Sr_2FeO_{3.875}$ (-350.67/-351.03eV) and Sr_2FeO_4 (-357.16eV). The vacancy formation is calculated relative to O_2 molecule (-8.22 eV) with the binding energy of -5.16 eV.
***VASP free energies are given solely for the reference. Relevant only with a particular set of computational parameters.
****The neighbours absent in the model supercell due to the periodic boundary conditions.

Defect interaction and formation energy analysis.

Fig. S 4 shows that the $V_O^{\bullet\bullet}$-$La_{Sr}^{\bullet}$ interaction energy has only a minor variation with $V_O^{\bullet\bullet}$-$La_{Sr}^{\bullet}$ distance. The only exception is the configuration in which the O from a La-O bond is removed (1NN), and the direct contact of the two $V_O^{\bullet\bullet}$ and $La_{Sr}^{\bullet}$ positive defects leads to a specific energy increase.

The $V_O^{\bullet\bullet}$ formation energy amounts to 2.35 eV for the lowest $V_O^{\bullet\bullet}$-in-FeO_2 configuration, and 2.60 eV for the lowest $V_O^{\bullet\bullet}$-in-(Sr,La)O configuration. These values are slightly higher (but still lagrely comparable to the respective values of 2.00 eV and 2.35 eV [2] in absence of La, although the initial iron oxidation state is 3.875+ instead of 4+. This is in line with the fact that for $Sr_2FeO_{4-\delta}$ only a minor variation of $V_O^{\bullet\bullet}$ formation energy with iron oxidation state has been observed[2].

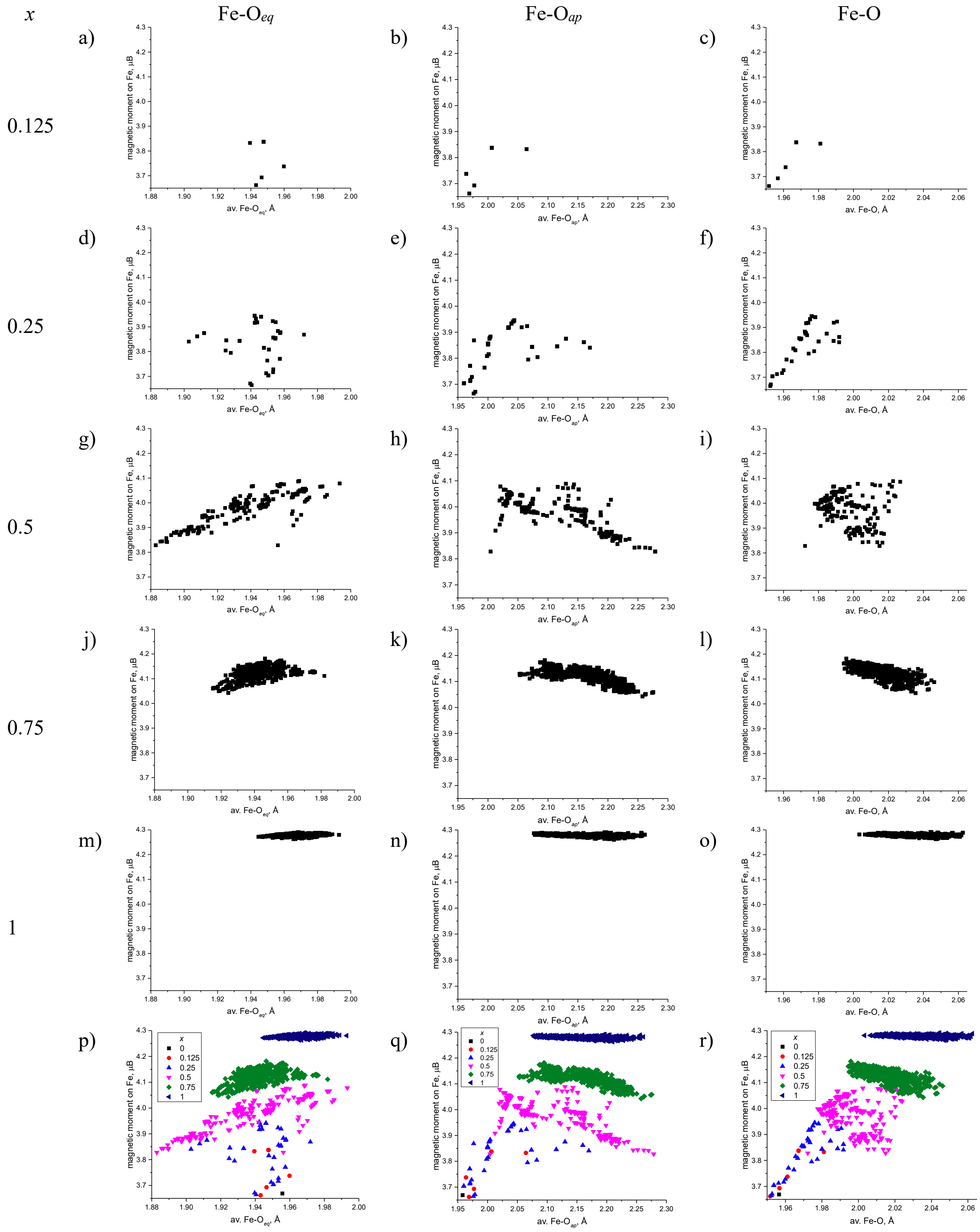

Figure S5. The plots of magnetic moment vs. Fe-O$_{ap}$, Fe-O$_{eq}$, and all Fe-O averaged over Fe cation bond lengths.

Table S5. Magnetic moment on Fe cations in $Sr_{2-x}La_xFeO_4$ and $Sr_2FeO_{4-\delta}$ for the configurations with the lowest energy. The excess electron charge for *x* corresponds to that for δ=*x*/2.

x(2δ)	Magnetic moment, μB	Oxidation state	$Sr_{2-x}La_xFeO_4$	$Sr_2FeO_{4-\delta}$
	Averaged formal		Calculated magnetic moment (with weights), μB	
0	4	4+	3.67	
0.125	4.125	3.875+	3.67×0.25; 3.74×0.125; 3.84×0.625	-
0.25	4.25	3.75+	3.81×0.25;3.86×0.5; 3.88×0.25	3.69×0.5; 3.90×0.25; 4.04×0.25
0.5	4.5	3.5+	3.88×0.5; 4.05×0.5	3.76×0.5; 4.13×0.5
0.75	4.75	3.25+	4.10×05; 4.12×0.5	-
1	5	3+	4.27	4.24×0.5; 4.25×0.5

Exploring a possible Fe^{3+} polaron formation depending on the starting conditions

The appearance of polarons as potentially only local energy minima may depend on the starting conditions of the energy minimization. This is explored below for the example of $x = 0.125$ (i.e. formally one Fe^{3+} in the supercell). It is deliberately done only for $x = 0.125$ because the lowest La content comes with the highest chance of obtaining localized electronic defects (polarons) at all. Even there, in the most localized state the single excess electron is not localized to a single iron, but distributed over five Fe cations (Table S 6). This indicates that strict localization of electrons as Fe^{3+} polarons is not feasible in the $Sr_{2-x}La_xFeO_4$ system, in which $V_O^{\bullet\bullet}$ which cause strong local distortions are absent.

Table S6. Averaged over all four Oeq anions, Fe-Oeq bond length in Å, and magnetic moment on Fe cation in μB. Fe cations are arranged by the distance from $La_{Sr}^{\bullet}$. For DFT+*U* the initial geometry was distorted around a single Fe cation (1-4NN), not distorted with all Fe-O*eq* bonds, having the same length (No JT), distorted with short and long Fe-O*eq* bonds (JT). For HSE06 calculations the initial geometry was JT-distorted. VASP free energies are given solely for comparison reason, and are valid only for the particular set of calculation parameters.

Fe-$La_{Sr}^{\bullet}$, NN	DFT+*U*												HSE06	
	1NN		2NN		3NN		4NN		No JT*		JT*			
	Fe-O*eq*	μ, μB	Fe-O*eq*	μ, μB	Fe-O*eq*	μ, μB	Fe-O*eq*	μ, μB	Fe-O*eq*	μ, μB	Fe-O*eq*	μ, μB	Fe-O*eq*	μ, μB
1	1.94	3.80	1.95	3.80	1.94	3.79	1.95	3.80	1.95	3.80	1.95	3.84	1.93	3.88
1	1.94	3.80	1.95	3.80	1.94	3.79	1.95	3.80	1.95	3.80	1.95	3.84	1.93	3.88
1	1.94	3.80	1.95	3.80	1.94	3.79	1.95	3.80	1.95	3.80	1.95	3.84	1.93	3.88
1	1.94	3.80	1.95	3.80	1.94	3.79	1.95	3.80	1.95	3.80	1.95	3.84	1.93	3.88
2	1.92	3.82	1.92	3.83	1.92	3.83	1.92	3.83	1.92	3.83	1.94	3.83	1.92	3.73
3	1.95	3.76	1.95	3.76	1.97	3.87	1.95	3.76	1.95	3.76	1.96	3.74	1.93	3.66
3	1.95	3.76	1.95	3.76	1.94	3.69	1.95	3.76	1.95	3.76	1.94	3.66	1.93	3.65
4	1.95	3.78	1.95	3.79	1.95	3.73	1.95	3.79	1.95	3.79	1.95	3.69	1.93	3.68
VASP E, eV	-363.075		-363.076		-363.079		-363.075		-363.076		-363.080		N/A	

Table S7. Structural and electronic parameters of $Sr_{2-x}La_xFeO_4$ depending on *x*. HSE06 exchange-correlation functional.

x		0 [2]	0.125*	0.25*	0.50*	0.75*	1.00*
a_0, Å		5.48	5.45	5.44	5.42	5.39	5.44
c_0, Å		12.36	12.46	12.57	12.82	13.00	12.95
volume/f.u., Å^3		92.89	92.47	93.00	94.06	94.41	95.81
Avg. bond length, Å	Fe-O(-Fe)	1.83/2.04****	1.93	1.93	1.92	1.92	1.95
	Fe(-O-Sr) ***	1.92	1.93	1.94	1.96	1.93	
	Fe(-O-La) ***	-	1.92	1.89	1.89	1.92	1.95
	Fe-O(-Sr)	1.92	1.96	1.95	1.97	1.99	1.93
	(Sr-)O-Fe(-O-Sr)	1.92	1.96	1.95	1.99	2.15	
	(Sr-)O-Fe(-O-La)		1.92	1.94	1.94	1.94	1.93
	Fe-O(-La)		2.03	2.44	2.52	2.53	2.54
μ**, μB	Fe	3.63	3.78	3.84	3.95	4.12	4.26
band width*****, eV	e_g	2.02	2.11	2.06	1.79	1.81	1.71
	t_{2g}	0.65	0.62	0.59	0.59	0.46	0.58
	(Sr/La-)O(-Fe)	4.28	5	4.84	4.6	4.9	5.48
	(Fe-)O(-Fe)	5.45	6.2	6.21	5.9	6.33	6.52
c.o.g.******, eV	e_g	-5.98	-6.51	-6.37	-5.93	-6.12	-6.52
	t_{2g}	-6.48	-7.07	-7.03	-6.52	-6.72	-7.09
	(Sr/La-)O(-Fe)	-2.39	-2.95	-2.78	-2.30	-2.64	-3.30
	(Fe-)O(-Fe)	-3.22	-3.93	-3.88	-3.60	-3.86	-4.21

*Configuration with the lowest energy.

**Averaged for all *x*>0.

*** Fe-O(-Fe) bond length for Sr- only or La-coordinated Fe. This is not Fe-O(-*A*) bond.

**** short and long Fe-O bonds in Sr_2FeO_4.

*****For Fe the meaningful band width was calculated as the width of the spin-up density within the rtange of [-9;-6] eV. For (Sr/La-)O(-Fe) – the Fermi level down to the first significant drop. For (Fe-)O(-Fe) – from the Fermi level down to the sharp edge at ~-7-5eV.

******The Centre of gravity was calculated from the Fermi level down to the full drop at ~-9ev.

Effects of DFT+*U* versus HSE06 functional on the degree of localization

To investigate the potential effect of DFT+*U* versus hybrid functionals, HSE06 calculations were performed for the lowest-energy configurations with $0.125 \leq x \leq 1$. Results are summarized in Table S 7, and compared to the corresponding DFT+*U* data (Table S 8).

Table S8. Structural and electronic parameters of $Sr_{2-x}La_xFeO_4$ depending on x. DFT+*U*, PBE exchange-correlation functional.

x		0 [2]	0.125*	0.25*	0.50*	0.75*	1.00*
a_0, Å		5.53	5.51	5.49	5.47	5.47	5.52
c_0, Å		12.47	12.58	12.68	12.84	12.96	12.86
volume/f.u., Å^3		95.39	95.33	95.60	96.04	96.99	98.05
Avg. bond length, Å	Fe-O(-Fe)	1.88/2.03	1.95	1.94	1.94	1.95	1.97
	Fe(-O-Sr) ***	1.96	1.95	1.95	1.97	1.95	-
	Fe(-O-La) ***	-	1.94	1.91	1.90	1.94	1.97
	Fe-O(-Sr)	1.96	1.99	2.00	2.03	2.01	1.96
	(Sr-)O-Fe(-O-Sr)	1.96	1.99	2.00	2.04	2.13	-
	(Sr-)O-Fe(-O-La)	-	1.98	2.00	2.03	1.97	1.96
	Fe-O(-La)	-	2.15	2.32	2.36	2.45	2.45
μ**, μB	Fe	3.67	3.78	3.85	3.97	4.12	4.26
band width, eV	e_g	1.96	1.87	1.98	1.98	1.83	1.97
	t_{2g}	0.54	0.71	0.47	0.58	0.43	0.53
	(Sr/La-)O(-Fe)	3.32	3.93	3.88	3.88	3.88	3.87
	(Fe-)O(-Fe)	4.93	5.26	5.21	5.19	5.36	5.24
c.o.g., eV	e_g	-5.82	-6.12	-5.80	-5.68	-5.58	-6.20
	t_{2g}	-5.67	-6.00	-6.00	-6.00	-6.12	-6.88
	(Sr/La-)O(-Fe)	-1.75	-1.95	-1.87	-1.75	-1.91	-2.82
	(Fe-)O(-Fe)	-2.54	-2.89	-2.85	-2.90	-3.00	-3.64

*Configuration with the lowest energy.
**Averaged for all $x>0$.
*** Fe-O(-Fe) bond length for Sr- only or La-coordinated Fe. This is not Fe-O(-*A*) bond.
**** short and long Fe-O bonds in Sr_2FeO_4.

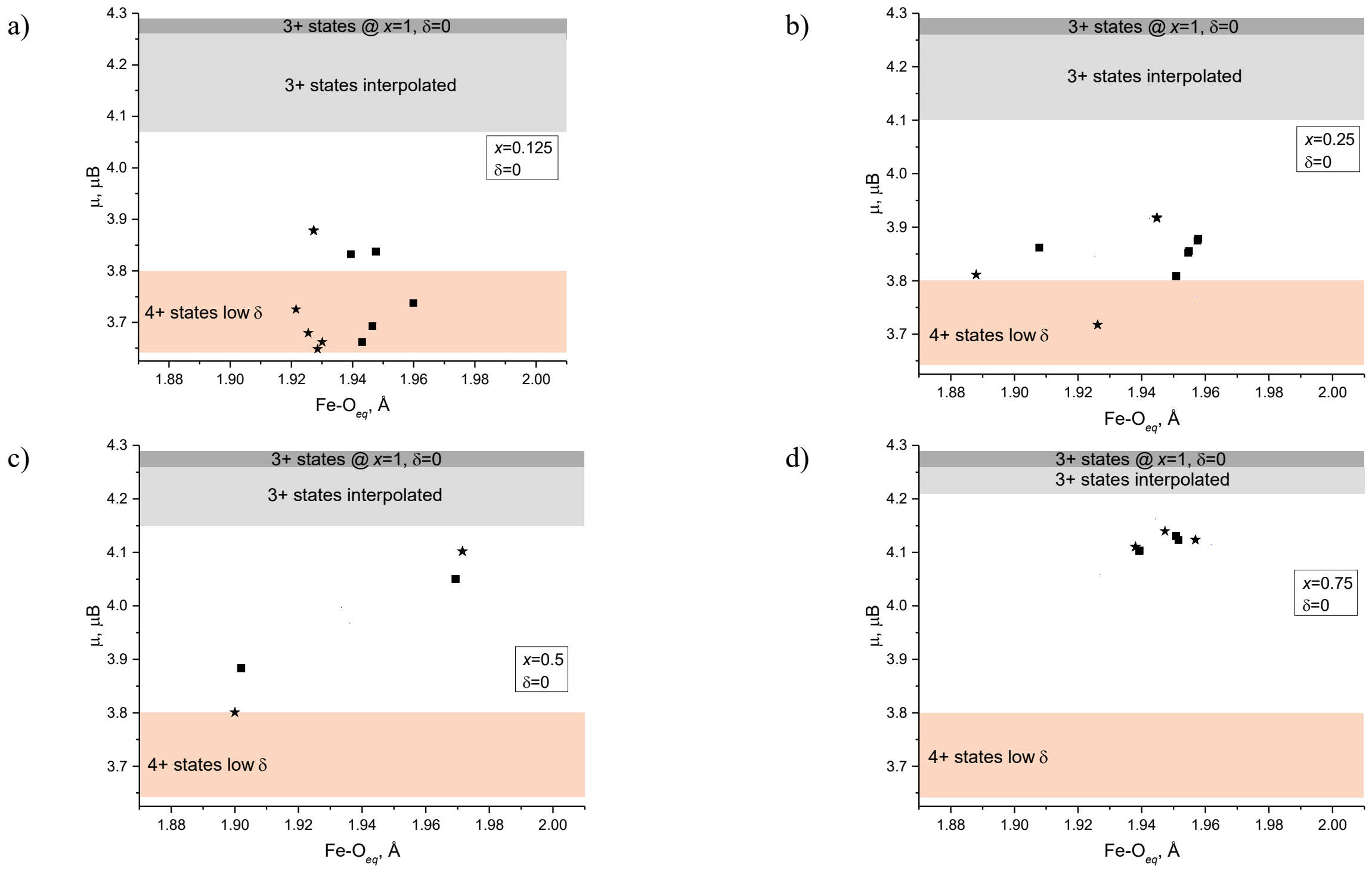

Figure S6. Magnetic moment as function of averaged Fe-O$_{eq}$ distance (average of the Fe-O$_{eq}$ bond length around each individual Fe in the supercell) for δ=0 and (a) x = 0.125, (b) x = 0.25, (c) x = 0.5, (d) x = 0.75. Square symbols=DFT+*U*, star symbols=HSE06.

Fe magnetic moment distributions for different La contents; dependence on Fe–O_{eq} distance; comparison of HSE06 and DFT+*U* results.

Fig. S6 shows the plots of magnetic moments of the individual Fe cations in the lowest-energy configurations for calculated with HSE various La contents as function of the averaged Fe-O_{eq} distance, analogously to Fig. 4 in the main text. For $x = 0.125$ and $x = 0.25$ a similar degree of localization is obtained by the HSE06 functional as for DFT+*U*. For $x = 0.125$ the data point at the highest magnetic moment actually comprises four/five degenerate Fe, i.e. the excess electron is shared among 4/5 iron atoms. For $x = 0.25$ the point of the highest magnetic moment represents an electron shared between two Fe. So also with HSE06 no strictly localized Fe^{3+} polarons are found. For $x = 0.5$ HSE06 yields a bimodal distribution of magnetic moments, which might appear as having four localized Fe^{3+} and four Fe^{4+} states. However, the magnetic moments still differ significantly from the reference values of 3.63 μ_B for Sr_2FeO_4 (only Fe^{4+} in the cell, Table S 7) and 4.26 μ_B for $LaSrFeO_4$ (only Fe^{3+} in the cell), questioning the assignment of integer oxidation states. For $x = 0.75$, the DFT+*U* and HSE06 results agree again quite well, both methodologies indicating a largely delocalized state (no real Fe^{3+} with magnetic moment ≥4.1 μ_B can be identified). This comparison indicates that while DFT+*U* yield some subtle differences, the overall picture remains the same.

Electronic density of states (DFT+*U* and HSE06) for $Sr_{2-x}La_xFeO_4$: electronic structure evolution with La content.

Electronic density of states DOS for the lowest-energy configurations with $0 \leq x \leq 1$ ($x = 0$ data taken from [2]), for DFT+*U* (U_{eff} = 4eV) as well as for HSE06. Figs. S 7 and S 8 show that the densities of state from both approaches have a very high similarity. They comprise the following key features for both DFT+*U* as well as for HSE: (i) Fe spin-up t_{2g} orbitals at low energy (ca. -6.5 eV for DFT+*U*, -7.5 eV for HSE06) and small band width. Generally, the splitting between spin-up and spin-down states is a bit larger in HSE compared to DFT+*U*. (ii) a maximum of the Fe e_g orbitals with a larger width at -6 to -8 eV (DFT+*U*) and -7 to -9 eV (HSE06). The Fe e_g orbitals strongly overlap with O 2*p* states. (iii) The DOS at -5 eV up to the Fermi level is dominated by O 2*p* states. (iv) Band gaps are observed in a quite similar matter for both functionals. They are seen most clearly for the high-symmetry configurations of $x = 0$, $x = 0.5$, $x = 1$ (for some intermediate La contents, the DOS is small but not exactly zero at the Fermi level, which might be related to local low-symmetry relaxations). The width of the band gap tends to be a bit smaller in DFT+*U*. So overall both DFT+*U* and HSE06 yield a rather similar picture, and the changes in the DOS with increasing La content are small.

To investigate whether the excess electrons introduced by $La_{Sr}^{\bullet}$ or $V_O^{\bullet\bullet}$ are localized or delocalized, the element-resolved DOS provides a reliable overall picture of the electronic structure and already captures the general localization trends in the system as a whole. In the $Sr_{2-x}La_xFeO$ materials studied here, there is no full localization into distinct Fe^{3+} sites. Even in the more localized $Sr_2FeO_{4-\delta}$ systems discussed previously [2], individual signatures of reduced Fe species are not straightforward to resolve in the element-projected DOS, which averages over all eight Fe atoms in the supercell.

A more detailed analysis of the specific spatial arrangement of localized charges would require atom-resolved projections and a substantially more elaborate investigation, which is beyond the scope of the present work. Therefore, local observables such as the magnetic moments of individual Fe atoms (Fig. 4 and 5) are used as a more suitable and sensitive descriptor for discussing variations in excess electron (de-)localization and its dependence on the compensating defects $La_{Sr}^{\bullet}$ or $V_O^{\bullet\bullet}$.

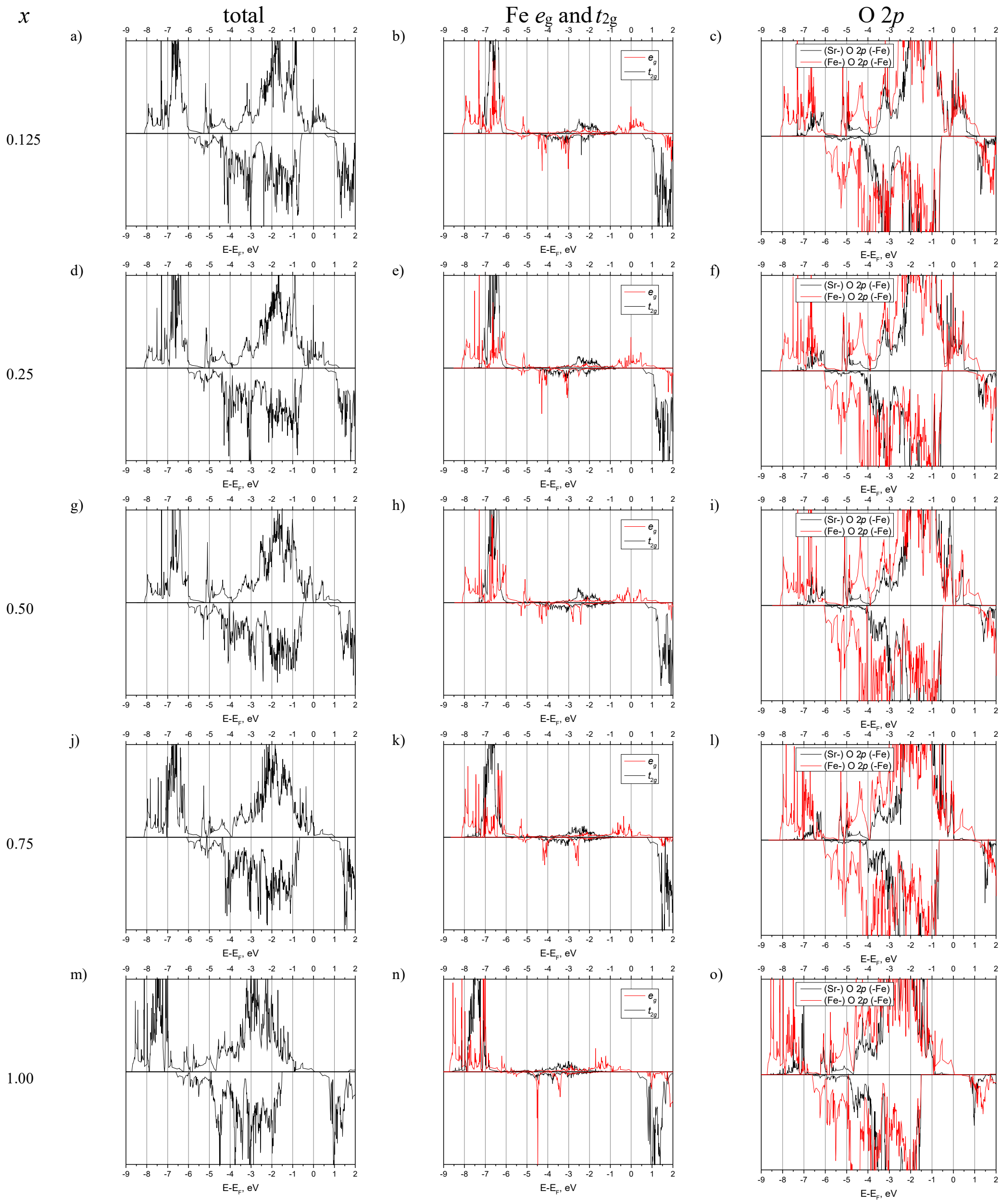

Figure S7. DOS from DFT+U for lowest-energy configurations with $0 \leq x \leq 1$.

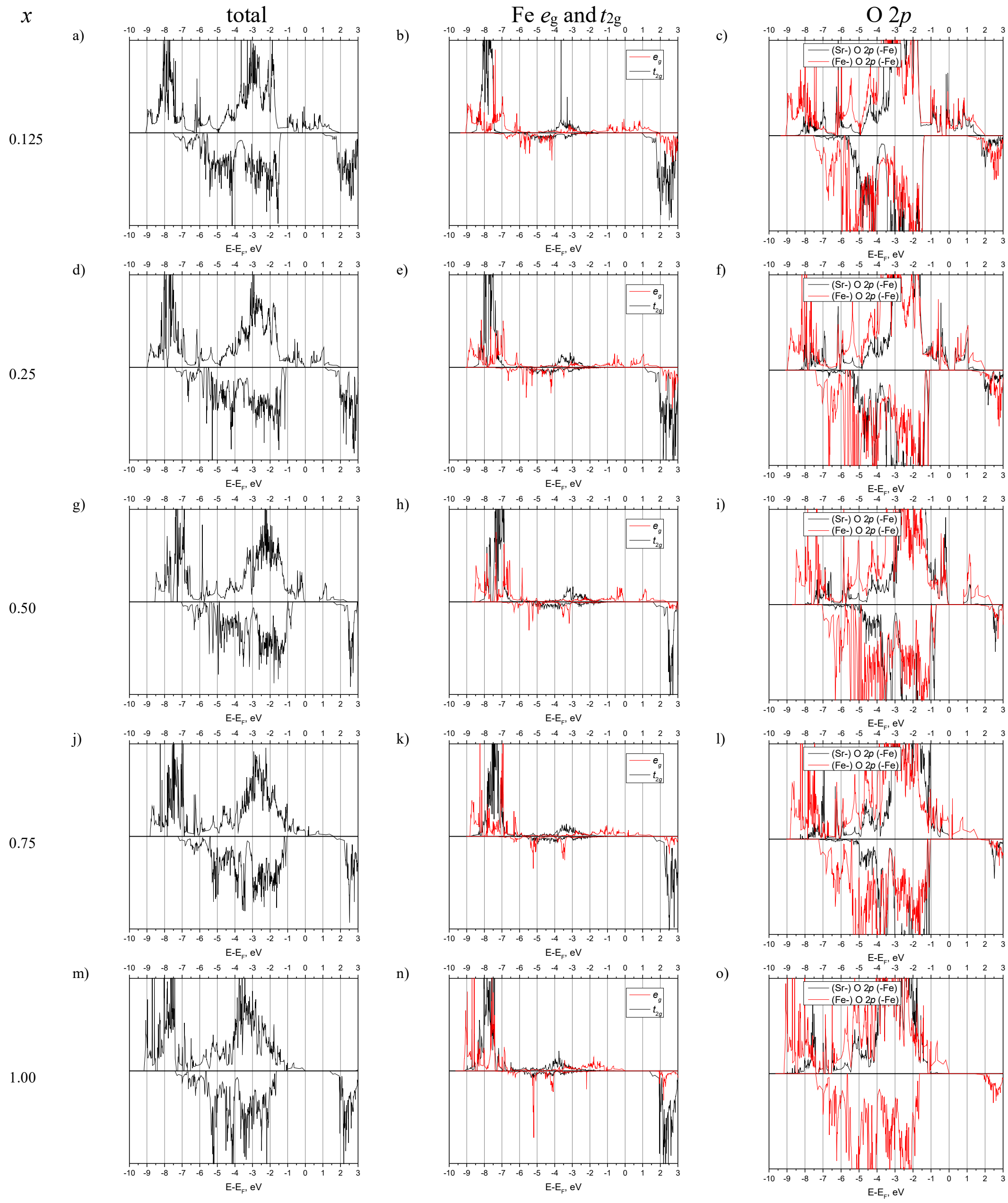

Figure S8. DOS from HSE06 for lowest-energy configurations with $0 \leq x \leq 1$.

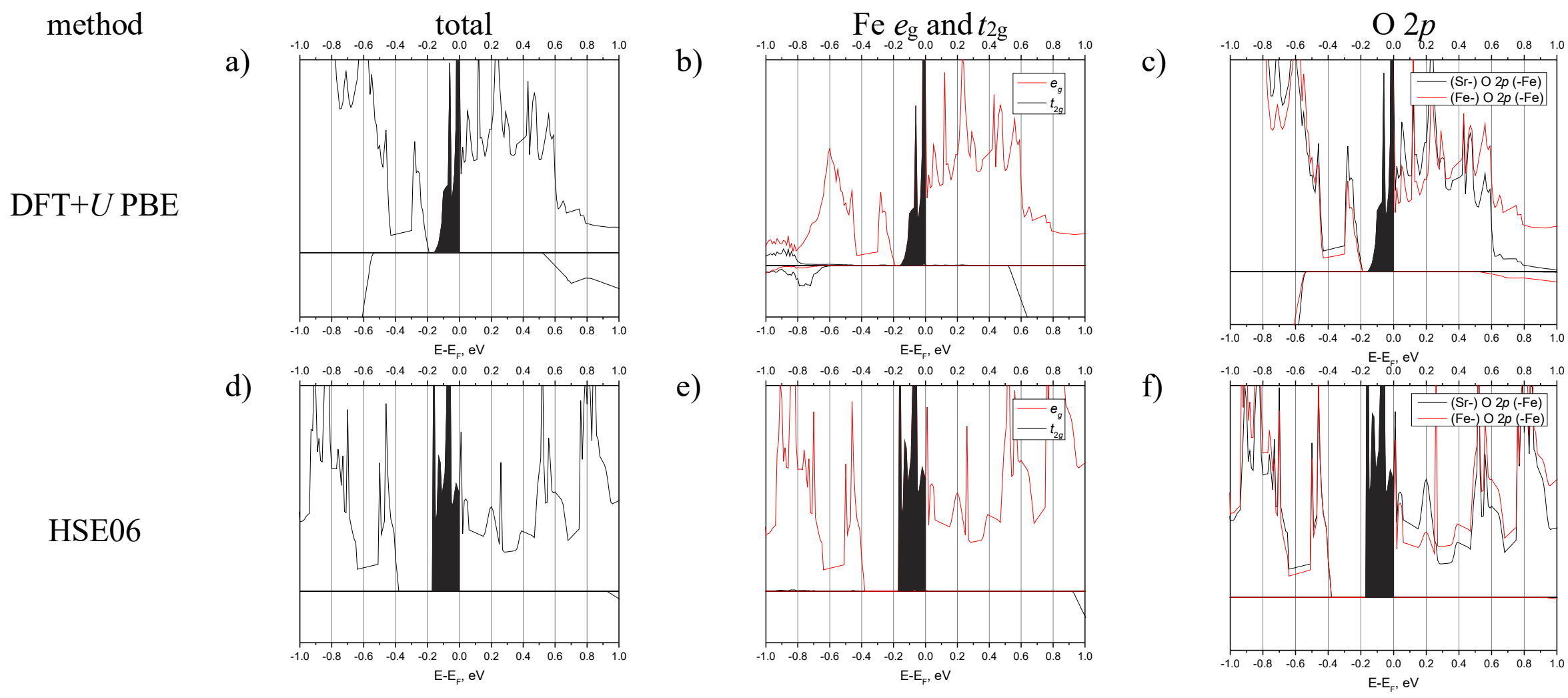

Figure S9. Zoom-in of DOS for $x = 0.125$. Filled area denotes the extra electron localized primarily on Fe 4×1NN and 2NN of La_{Sr}.
